# Immunosuppressive properties of *Pluchea lanceolata* leaves

**DOI:** 10.4103/0253-7613.62405

**Published:** 2010-02

**Authors:** D.P. Bhagwat, M.D. Kharya, Sarang Bani, Anpurna Kaul, Kiranjeet Kour, Prashant Singh Chauhan, K.A. Suri, N.K. Satti

**Affiliations:** Pharmacology Research Laboratory, ASBASJSM College of Pharmacy, Bela (Ropar), Punjab, India; 1 Department of Pharmaceutical Sciences, Dr. Hari Singh Gour University, Sagar, MP, India; 2 Cell Biology Laboratory, Division of Pharmacology, Jammu Tawi, J and K, India; 3 Natural Products Chemistry Division, Indian Institute of Integrative Medicine, Jammu Tawi, J and K, India

**Keywords:** CD 4 T-helper cells, CD 8 T-cytotoxic cells, cell-mediated immune response, Humoral antibody titer, *Pluchea lanceolata*.

## Abstract

**Objective:**

To investigate the immunosuppressive potential of Pluchea lanceolata 50% ethanolic extract (*PL*) and its bioactive chloroform fraction (*PLC*).

**Materials and Methods:**

Preliminary screening of the *Pluchea lanceolata* 50% ethanolic extract (*PL*) was carried out with basic models of immunomodulation, such as, the humoral antibody response (hemagglutination antibody titers), cell-mediated immune response (delayed-type hypersensitivity), skin allograft rejection test, *in vitro* (*C. albicans* method), and *in vivo* phagocytosis (carbon clearance test).

The extract was then fractionated with chloroform, n-butanol, and water to receive the respective fractions by partitioning. These fractions were employed for flow cytometry to study the T-cell specific immunosuppressive potential of these fractions.

**Results:**

Oral administration of PL at doses of 50 to 800 mg/kg in mice, with sheep red blood cells (SRBC) as an antigen, inhibited both humoral and cell-mediated immune responses, as evidenced by the production of the circulating antibody titer and delayed-type hypersensitiviy reaction results, respectively, and the immune suppression was statistically significant (*P* < 0.01) in Balb/C mice. PL also decreased the process of phagocytosis both *in vitro* (31.23%) and *ex vivo* (32.81%) and delayed the graft rejection time (30.76%). To study the T-cell-specific activities, chloroform, n-butanol, and water fractions from *P. lanceolata* were tested for T-cell specific immunosuppressive evaluation, wherein only the chloroform fraction (PLC) showed significant (*P* < 0.01) suppression of CD8+ / CD4+ T-cell surface markers and intracellular Th1 (IL-2 and IFN-_Y_) cytokines at 25 – 200 mg/kg p.o. doses. PLC, however, did not show significant suppression of the Th2 (IL-4) cytokine.

**Conclusion:**

The findings from the present investigation reveal that *P. lanceolata* causes immunosuppression by inhibiting Th1 cytokines.

## Introduction

In the present study, we have investigated the immunosuppressive properties of *Pluchea lanceolata* C.B. Clarke (Asteraceae) popularly known as ‘Rasana’. It is widely used in the treatment of rheumatoid arthritis in the indigenous systems of medicine.[1] This plant is indicated in Ayurvedic texts for its therapeutic usefulness in diseases similar to rheumatoid arthritis and other afflictions of joints. Tribally, a poultice of leaves is applied to the inflamed areas of the body. The Ayurvedic practitioners of the region use this drug for treating pain and swelling of the body joint.[2,3] In experimental arthritis, a decoction of the plant has been reported to prevent the swelling of joints.[4] The leaves are aperient and used as a laxative, analgesic, and antipyretic. Quercetin and isorhamnetin have been identified in the air-dried leaves of *P. lanceolata*.[5,6] Anti-inflammatory activity has been reported in the neolupinol isolated from the flowers of this plant.[7] Flavonoids are reported as scavengers of free radicals[8,9] and potent inhibitors of lipid peroxidation. Oral pre-treatment of the ethanolic extract significantly attenuated cadmium chloride, induced oxidative stress, and genotoxicity.[10]

In the present study, we have investigated the immunosuppressive properties of *P. lanceolata* exxtract (*PL*) and its corresponding chloroform fraction (*PLC*), as also its plausible role in relieving the inflammatory conditions of the body.

## Materials and Methods

### Plant material

*Pluchea lanceolata* leaves were collected from the local market at Sagar, a city in Madhya Pradesh, India, and authenticated by the routine pharmacognostic procedures by Dr B. K. Kapahi, a senior scientist in the Botany Division of the Indian Institute of Integrative Medicine (IIIM), Jammu, India. A voucher specimen was retained and deposited at the crude drug repository of the herbarium of IIIM, vide CDR accession No. 3233.

### Preparation of extract and fractions

*Pluchea lanceolata* (*PL*) leaves (2.0 Kg) were macerated with ethanol:water (1:1), with constant stirring at 1200 rpm, at room temperature, for three hours. The solvent was filtered, marc was drained, and the procedure repeated thrice for complete extraction of the phytochemicals. The combined extracts were reduced to one-eighth of their original volume under rotavapor (Heidolph Laborota 4000), at 50°C, and lyophilized to get a yield of 295.5 g. This lyophilized extract was macerated with chloroform, and the chloroform-soluble portion was separated and evaporated on a rotavapor to obtain the chloroform fraction (70.67 g). The chloroform-insoluble portion was partitioned between n-butanol and water to obtain the n-butanolic (17.33 g) and water fraction (207.5 g), respectively.

### Animals

Male Balb/c mice (Mus musculus) 8-10 weeks old and weighing 18-22 g, in groups of six, were used for the study. The animals were housed under standard laboratory conditions with a temperature of 23 ± 1°C, relative humidity of 55 ± 10%, 12/12 hour light-dark cycles, and fed with a standard pellet diet (Lipton India Ltd) and water was given ad libitum. None of the animals were sacrificed throughout the study.

### Drugs

Drugs for oral administration were freshly prepared as a homogenized suspension of *Pluchea lanceolata* extract (*PL*) in doses of 50, 100, 200, 400, 600, and 800 mg/kg and *Pluchea lanceolata* chloroform fraction (*PLC*) in doses of 25, 50, 100, and 200 mg/kg in 1% w/v gum acacia and administered orally, once daily, for the duration of the experiment on Balb/C mice.

Cyclophosphamide and cyclosporine-A were used as the standard immunosuppressive agents at 250 and 5 mg/kg, p. o. dose for B-cell and T-cell suppression, respectively.

### Chemicals

Fluoroisothiocyanate (FITC)-labeled CD4 anti-mouse monoclonal antibody, Phycoerythrin (PE)-labeled CD8 anti-rat monoclonal antibody, FACS lysing solution, FACS permeabilising solution, Golgi plug, IL-2 monoclonal antibody anti-mouse (PE), IFN-γ- monoclonal antibody anti-mouse (FITC) (BD Biosciences); phosphate buffer saline, ethylene diamine tetraacetic acid (EDTA) (Sigma Aldrich), and heparin (Sigma Aldrich) were used. All other reagents used were of analytical grade.

### Antigenic stimulus

Fresh sheep red blood cells (SRBC) collected aseptically from the jugular vein of sheep were stored in cold sterile Alsever's solution and washed thrice with pyrogen-free sterile normal saline (0.9% NaCl w/v). Cell count was adjusted to 5 × 10^9^ cells/ml for immunization and challenge at the required time schedule.

### Blood collection

On the specified days, as mentioned in the experimental protocols, blood was drawn from the retro-orbital plexus of the animals and EDTA was used as an anti-coagulant.

### Effect on general behavior and acute toxicity in mice

The acute oral toxicity studies were carried out following the OECD guidelines (No.423).[11] Three female Balb/C mice were used for each step. The animals were fasted for three to four hours before the oral administration of the *PL* fraction and observed individually, after dosing, at least once during the first 30 minutes, periodically during the first 24 hours, with special attention given during the first four hours, and daily thereafter, for a total of 14 days. The animals were observed for general behavior and any toxic symptoms produced by the test materials under investigation, along with the routine pharmacological parameters, such as, cyanosis, tremors, convulsions, ataxia, body tone, muscle tone, piloerection, salivation, tail flick, drowsiness, alertness, spontaneity, diarrhea, pupil size, ptosis, breathing rate, urination, and so on. The animals did not show any toxic and abnormal symptoms up to the single oral dose of 2000 mg/kg.

### Selection of doses

No toxic effect was observed in mice up to 2000 mg/kg *p.o.* dose (LD_0_). We took one-tenth of this dose (200 mg/kg, *p.o.*) with one lower (100 mg/kg) and one higher (400 mg/kg) dose for activity evaluation. On biological evaluation, we found the effect to be significantly increased at the 400 mg/kg dose, and therefore, we then took higher doses (600 and 800 mg/kg), to get to a dose that would have the maximum immunomodulatory effect. The basic experiments for immunosuppressive studies, such as, humoral antibody response, delayed type hypersensitivity, skin allograft rejection test, and phagocytosis were performed with *P. lanceolata* 50% EtOH extract (*PL*) and further studies on flow cytometry were performed on fractions by partitioning the extract with chloroform, n-butanol, and water to separate the polarity-based phytoconstituents.

### Statistical analysis

Data were expressed as the mean ± standard error of the means (S.E.), and statistical analysis was carried out employing the Dunnett test, which compares the test groups and standard drug group with the control group.

### Experimental protocols

All experimental protocols and the number of animals used for the experimental work were duly approved by the Institutional Animals Ethics Committee (IAEC) of Dr. H.S. Gour University, Sagar, M. P. (CPCSEA registration No. 379/01/ab/CPCSEA; vide approval No. Animal Eths.Comm./DB/98, dated 29.07.2006.

### Humoral antibody response

The mice were immunized by injecting 200 μl of 5 × 10^9^ SRBC/ml intraperitoneally (*i.p.*) on day 0. *Pluchea lanceolata* 50% Ethanol extract (*PL*) was administered to the mice in graded doses (50, 100, 200, 400, 600, and 800 mg/kg body weight, *p.o.*) for seven days (Group III – VIII). Hemagglutination primary and secondary antibody titers were performed following the method described by Nelson and Mildenhall.[12] Bovine serum albumin-saline (BSA-saline) served as the control (Group I) and cyclophosphamide (250 mg/kg) was administered once only on day 0 of the experiment as a standard immunosupressor (Group II).

### Delayed-type hypersensitivity response (DTH)

PL was administered to mice after SRBC sensitization and once daily for seven days (Group III – VIII) and cyclosporine-A (5 mg/kg) was administered as the standard T-cell suppressor (Group II). Group I served as the sensitized control. The mice were then challenged by injecting the same amount of SRBC intradermally into the right hind footpad, whereas, the left footpad served as a control. The foot thickness was measured with a spheromicrometer (pitch, 0.01 mm) at 0 and 24 hours of SRBC challenge.[13]

### Skin allograft rejection

An equal pinch of homologous graft of skin was transplanted in groups of six inbred mice as per the method described by Billingham and Medawar. PL was administered for seven days and the graft rejection time was recorded by daily observations of the epithelial skin layer survival. The negative control group was administered vehicle and the positive control group received cyclosporine-A (5 mg/kg) daily for seven days.[14]

### Phagocytic response in vitro

The method of Lehrer was used for opsonization of peritoneal macrophages with heat killed *Candida albicans* cells and the percentage and average number of *C. albicans* cells (heat killed) ingested by peritoneal murine macrophages were calculated.[15]

### Phagocytic response ex vivo

Groups of six mice were injected (*i.v.*) 160 mg/kg of 1.6% suspension of gelatin-stabilized carbon particles, of size 20–25 μm.[16] Blood samples were collected before and at intervals varying between 2 to 90 minutes after carbon injection. Aliquots (10 μl) of blood samples were lysed with 2 ml of 0.1% acetic acid and the transparency determined spectrophotometrically at 675 nm[17] (Uvikon 810, spectrophotometer, Kontron Ltd., Switzerland).

### Lymphocyte immunophenotyping

*PL* was fractionated by partitioning with chloroform, n-butanol, and water, to separate the non-polar and polar phytoconstituents, for assessing the respective bioactivities. Out of these three fractions, only the chloroform fraction of *P. lanceolata* (*PLC*) showed potential bioactivity, Hence it was explored and discussed in all flow cytometric estimations. For estimating the T-cell surface markers, the method of Bani et al., was used.[18] Analysis of the recorded data was carried out by using the Cell Quest Pro software on a BD LSR-II flow cytometer (BD Biosciences, USA).

### Method for intracellular cytokine estimation

For estimating intracellular Th1 cytokines, such as, interleukin-2 (IL-2), interferon-_γ_ (IFN-_γ_), and Th2 cytokine interleukin-4 (IL-4), the method of Bani et al.,[19] was used.

## Results

### Humoral antibody response

*PL* between doses 50 to 800 mg/kg *p.o.*, produced a dose-related decrease in the primary and secondary antibody formation with the maximum decrease of 33.38 and 30.03%, respectively, at a dose of 600 mg/kg p.o., after which the suppressive effect waned. It was 25.69% (primary) and 27.47% (secondary), respectively, at the 800 mg/kg dose. Cyclophosphamide (250 mg/kg) was used as a standard drug and showed 36.00% decrease in antibody formation at the primary and 34.98% decrease at the secondary antibody titers [Table 1]. This indicates that *PL* decreases primary and secondary antibody production significantly (*P* < 0.01). However, the effect was slightly more on primary antibody formation [Table 1].

**Table 1:** Effect of *Pluchea lanceolata (PL)* on SRBC-induced primary (Habt) and secondary (Sabt) humoral immune response and delayed type hypersensitivity (DTH) response (CMI) in mice

*Treatment dose (mg/kg p.o.)*	*Humoral immune response*				*Cell-Mediated immune response*	
		
	*Habt [log_2_] day 7*		*Sabt [log_2]_day 14*		*Paw swelling (mm) in mice day 7*	
			
	*(Mean ± S.E.)*	*%Change*	*(Mean ± S.E)*	*%Change*	*(Mean ± S.E)*	*%Change*
Control SRBC	6.50 ± 0.22	-	6.66 ± 0.33	-	0.71 ± 0.04	-
Cyclophosphamide(250)	4.16 ± 0.21**	36.00↓	4.33 ± 0.01**	34.98↓	-	-
Cyclosporine-A (5)	-	-	-	-	0.31 ± 0.02**	56.33 ↓
*PL* (50)	6.16 ± 0.16**	5.23 ↓	6.33 ± 0.21	4.95 ↓	0.53 ± 0.09	25.35↓
*PL* (100)	5.66 ± 0.21**	12.92 ↓	5.83 ± 0.16*	12.46 ↓	0.48 ± 0.09*	32.39 ↓
*PL* (200)	5.33 ± 0.21**	18.00 ↓	5.50 ± 0.22**	17.41 ↓	0.45 ± 0.05*	36.61 ↓
*PL* (400)	5.00 ± 0.25**	23.07 ↓	5.16 ± 0.16**	22.52 ↓	0.40 ± 0.02**	43.66 ↓
*PL* (600)	4.33 ± 0.21**	33.38 ↓	4.66 ± 0.21**	30.03 ↓	0.35 ± 0.02**	50.70 ↓
PL (800)	4.83 ± 0.30**	25.69 ↓	4.83 ± 0.16**	27.47 ↓	0.37 ± 0.02**	47.88 ↓

* *P* < 0.05;

** *P* < 0.01 (Dunnett test which compares the test groups and standard drug groups with the control group); Habt, humoral antibody titer (primary); Sabt, secondary antibody titer; ↓ reduction in activity; number of animals per group = 6; CMI = cell-mediated immunity.

### Delayed-type hypersensitivity response (DTH)

*PL* (50–800 mg/kg, *p.o.*) showed a decrease of 25.35 – 50.70% in DTH response in mice. The most significant effect was observed at 600 mg/kg dose (*P* < 0.01). The effect of PL at 800 mg/kg reduced to 47.88%. Cyclosporine at 5 mg/kg dose produced a decrease of 56.33% in DTH response [Table 1].

### Skin allograft rejection

*PL* at 50–800 mg/kg delayed the skin allograft rejection time (days) in mice. The maximum delay in rejecting the graft was observed to be 30.76% at 800 mg/kg (*P* < 0.01). However, *PL* at doses of 50, 100, 200, 400, and 600 mg/kg delayed the graft rejection time by 2.53, 12.76, 17.92, 21.76, and 26.92%, respectively. Cyclosporine-A (5 mg/kg) delayed the graft rejection time by 57.69% [Table 2].

**Table 2:** Effect of *Pluchea lanceolata* (PL) on homologous graft rejection in mice

*Group*	*Treatment (mg/kg)*	*Rejection time (days) (mean ± S.E.)*	*% activity*
I	Naive control (NC)	13.00 ± 0.25	-
II	Cyclosporine-A (5)	20.50 ± 0.34**	57.69 ↑
III	PL(50)	13.33 ± 0.21	2.53 ↑
IV	PL(100)	14.66 ± 0.33**	12.76 ↑
V	PL(200)	15.33 ± 0.21**	17.92 ↑
VI	PL(400)	15.83 ± 0.40**	21.76 ↑
VII	PL(600)	16.50 ± 0.34**	26.92 ↑
VIII	PL(800)	17.00 ± 0.51**	30.76 ↑

** *P* < 0.01 (Dunnett test, which compares test groups and standard drug group with control group); number of animals per group = 6

### Phagocytic response in vitro

*PL* was administered to mice at the doses of 50-800 μg/ml. Significant reduction was observed at 400, 600, and 800 μg/ml doses, where the effect was found to be 21.52, 31.23, and 27.62%, respectively (*P* < 0.01). Cyclosporine-A at 10 μg/ml showed 45.46% decrease (*P* < 0.01) in phagocytosis of heat killed Candida albicans by murine macrophages [Table 3].

**Table 3:** Effect of *Pluchea lanceolata (PL)* on phagocytic function of murine macrophages

*Treatment(Dose μg/ml)*	% *Phagocytosis (in vitro study) mean ± S.E.*		*Treatment (dose mg/kg)*	*Phagocytic Index (in vivo study) (Mean ± S.E.)*	
Naïve Control	25.23 ± 1.26	-	Sensitized Control	1.28 ± 0.12	-
Cyclosporine-A(10)	13.76 ± 0.33**	(45.46) ↓	Cyclosporine-A(5)	0.69 ± 0.10**	(46.09) ↓
*PL* (50)	24.12 ± 1.33	(4.39) ↓	*PL* (50)	1.24 ± 0.14	(3.12) ↓
*PL* (100)	22.48 ± 0.92	(10.89) ↓	*PL* (100)	1.20 ± 0.10	(6.25) ↓
*PL* (200)	21.79 ± 0.75*	(13.63) ↓	*PL* (200)	1.12 ± 0.12*	(12.50) ↓
*PL* (400)	19.80 ± 0.75**	(21.52) ↓	*PL* (400)	1.00 ± 0.10**	(21.87) ↓
*PL* (600)	17.35 ± 0.59**	(31.23) ↓	*PL* (600)	0.84 ± 0.12**	(44.53) ↓
*PL* (800)	18.26 ± 0.39**	(27.62) ↓	*PL* (800)	0.83 ± 0.14**	(45.31) ↓

* *P* < 0.05;

** *P* < 0.01 (Dunnett test, which compares test groups and the standard drug group with the control group); Figures in parentheses represent percent change in activity; number of animals per group = 6

### Phagocytic response ex vivo

*PL* administered to mice for a period of seven days decreased the rate of clearance of carbon particles from the circulation [Table 3]. The decrease in phagocytic index was 3.12–45.31% at a dose range of 50 – 800 mg/kg. The effect was highly significant at higher doses (*P* < 0.01).

### Lymphocyte immunophenotyping

**Figure 1:**
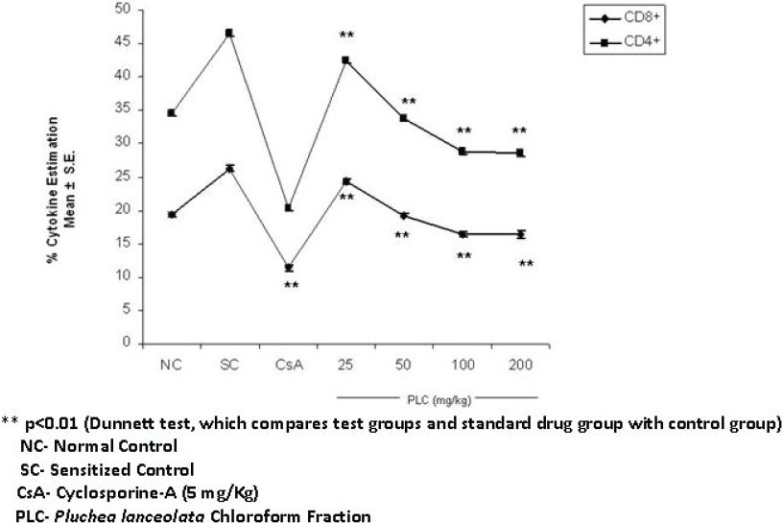
Effect of chloroform fraction of *Pluchea lanceolata* (PLC) on CD8+/CD4+ T-cell surface markers

Only the chloroform fraction of *P. lanceolata* (*PLC*) exhibited immunosuppressive activity and thus explored further for its immunosuppressive spectrum. *PLC* showed the maximum effect at 100 mg/kg p.o. dose, which accounts for 16.37% (Mean ± S.E.) of CD8+ and 28.45% (Mean ± S.E.) of CD4+ T-cells (*P* < 0.01). The control values were 26.27% (Mean ± S.E.) of CD8+ and 46.5% (Mean ± S.E.) of CD4+ T-cells. This shows a significant suppression of CD8+ and CD4+ T-cell population (*P* < 0.01). Cyclosporine-A, a standard T-cell inhibitor at 5 mg/kg oral dose significantly (*P* < 0.01) inhibited both CD8+ and CD4+ T-cells, and showed 11.35% (Mean ± S.E.) of CD8+ and 20.31% (Mean ± S.E.) of CD4+ T cells [Figure 1].

### Intracellular cytokines estimation

**Figure 2:**
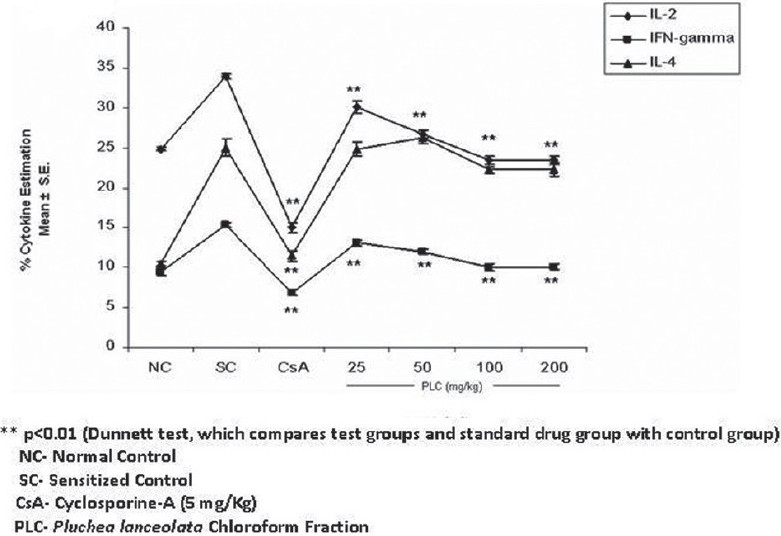
Effect of chloroform fraction of *Pluchea lanceolata* (PLC) on IL-2, IFN-γ, and IL-4

IL-2 and IFN-γ were assayed, as the change in the relative proportion of the lymphocyte subsets was proportional to the change in the cytokine expression patterns. Both IL-2 and IFN-γ counts were downregulated. The reduction in IL-2 by PLC was observed at all the dose levels, with maximum expression at a dose of 100 mg/kg, where it was found to be 23.40 ± 0.52 (% mean ± SE) in CD 4+ T cells (p < 0.01). The percentage of IL-2 in the sensitized control group was 33.93 ± 0.26 (% mean ± SE) and IFN-γ decreased from 15.36 ± 0.34 (sensitized control) to 10.10 ± 0.44 (% mean ± SE) in the PLC-treated group, at 100 mg/kg (p < 0.01). However, cyclosporine-A, which was used as a standard, significantly (p < 0.01) decreased IL-2 and IFN-γ by 15.03 ± 0.67 and 6.82 ± 0.34, respectively [Figure 2].

The percentage of IL-4 in the normal sensitized group was 25.00 ± 1.11 (mean ± S.E.). The maximum inhibition of IL-4 in the CD 4+ T cells was 22.22 ± 0.35 (mean ± S.E.) at 100 mg/kg dose p.o., which did not show a significant reduction [Figure 2]. The cyclosporine-A-treated group showed a significant (p < 0.01) decrease in IL-4 by 11.48 ± 0.67 (mean ± S.E.), at 5 mg/kg dose.

## Discussion

*Pluchea lanceolata* C.B. Clarke (Asteraceae), popularly known as ‘Rasana’ has been traditionally used since ancient times by Ayurvedic practitioners, to treat various painful afflictions of joints.[1,2] Therefore, it was decided to investigate the immunosuppressive potentials of this plant. The experimental design was carried out in two phases, where, in the first phase, the 50% ethanolic (EtOH) extract of *P. lanceolata* (*PL*) was explored for its immunosuppressive properties, using base models of immunomodulation, such as, humoral immune response, cell-mediated immune response (DTH), skin allograft rejection test, and *in vitro* and *in vivo* phagocytic responses, and in the second phase the 50% EtOH extract was fractionated by partitioning with chloroform, n-butanol, and water, to separate the polarity-based molecules and investigate them for their presence or absence of bio-activity. The 50% EtOH extract of *P. lanceolata* (*PL*) showed a significant decrease in the humoral and cell-mediated immune responses, *in vitro* and *in vivo* phagocytic bait. *PL* delayed the time for graft rejection, which again substantiated its putative immunosuppressive nature. The skin allograft rejection test is the simplest paradigm to assess cell-mediated immune response and is suggestive of the fact that *PL* has significant activity on cell-mediated immune responses also. Furthermore, downregulation of phagocytosis of murine macrophages also confirmed the immunosuppressive potential of *PL*. Macrophages play a vital role in immunity through cell-mediated immune responses. Macrophage activity is an indicator to prove the potential of an individual drug as an immunoenhancer or immunosuppressor. These results prompted us to fractionate 50% of the ethanolic extract and further extrapolate our studies on bioactive fractions. On further extrapolation of this study, the chloroform, n-butanol, and water fractions were investigated for flow cytometric studies on Th1 and Th2 cytokines, wherein, only the chloroform fraction (*PLC*) showed marked immunosuppressive and anti-inflammatory activity, as evidenced from the downregulation of T-cell surface markers (CD8+/CD4+) and intracellular Th1 (IL-2 and IFN-γ) cytokines [Figures 1 and 2]. Normally, T cells expressing CD4 increase when the physiological systems of the body are stimulated, due to the activation of the non-specific immune status, and inhibition of this phenomenon shows the immunosuppression. When the body's defense mechanism is threatened by microorganisms or any other foreign invaders, the macrophages reactivate at the site of invasion to combat establishment of various infections. CD 4+ T-cell inhibition by *P. lanceolata* may be one of the factors responsible for the decrease in the functioning of the macrophages. The reduced responsiveness of the phagocytes is evident by the decrease in the clearance of carbon particles from the reticuloendothelial system and also in the reduction in the rate of phagocytosis *in vitro* by murine macrophages, thereby, suggesting a reduction in the functioning of macrophages [Table 3]. *PLC* inhibited IL-2 production by CD4+ T cells in a dose-dependent manner [Figure 2]. It is a growth factor for Th1 cytokines. It stimulates and facilitates the biosynthesis of IFN- γ in T cells. The inhibition of IL-2 is possibly responsible for the reduced secretion of IFN- γ by CD 8+ T cells. Apart from its role in T-cell activation, IFN-γ is known to be central to the full-blown activation of macrophages[20] and its inhibition by *PLC* may be one of the important factors that cause a reduction in macrophage function. Generally, IFN- γ is released from T cells and macrophages, which proliferate the process of phagocytosis.[21,22] However, the significant reduction in IL-2 and IFN-γ is suggestive of the immunosuppressive nature of *PLC*. Moreover, *PLC* has not shown significant reduction in IL-4 expression, which suggests that *P. lanceolata* has little effect on the Th2 system.

## Conclusion

Th1/Th2 paradigm not only allows a better understanding of the main mechanisms involved in the protection and pathogenesis of several immunopathic disorders, but also provides the basis for cytokine-induced immune deviation.

*P. lanceolata* inhibited the humoral antibody response and cell-mediated immune responses. Flow cytometric studies also revealed the downregulation of pro-inflammatory cytokines and this is suggestive of its possible therapeutic usefulness in the treatment of the inflammatory states of the body and autoimmune disorders like arthritis. However, the clinical margin of safety in long-term therapeutics has to be established along with its biopharmaceutical evaluation for future therapeutic considerations.
